# Effect of Monkeypox Virus Preparation on the Lethality of the Intravenous Cynomolgus Macaque Model

**DOI:** 10.3390/v14081741

**Published:** 2022-08-09

**Authors:** Eric M. Mucker, Josh D. Shamblin, Jo Lynne Raymond, Nancy A. Twenhafel, Robert F. Garry, Lisa E. Hensley

**Affiliations:** 1 Virology Division, United States Army Medical Research Institute of Infectious Diseases, Fort Detrick, Frederick, MD 21702, USA; 2 Pathology Division, United States Army Medical Research Institute of Infectious Diseases, Fort Detrick, Frederick, MD 21702, USA; 3 Department of Microbiology and Immunology, School of Medicine, Tulane University, New Orleans, LA 70112, USA; 4 Zalgen Labs, Frederick, MD 21703, USA; 5 Global Virus Network (GVN), Baltimore, MD 21201, USA; 6 Zoonotic and Emerging Disease Unit, United States Department of Agriculture, Manhattan, KS 66505, USA

**Keywords:** orthopoxvirus, purified, nonhuman primates, viremia, model, smallpox, monkeypox, countermeasures, aggregation

## Abstract

For over two decades, researchers have sought to improve smallpox vaccines and also develop therapies to ensure protection against smallpox or smallpox-like disease. The 2022 human monkeypox pandemic is a reminder that these efforts should persist. Advancing such therapies have involved animal models primarily using surrogate viruses such as monkeypox virus. The intravenous monkeypox model in macaques produces a disease that is clinically similar to the lesional phase of fulminant human monkeypox or smallpox. Two criticisms of the model have been the unnatural route of virus administration and the high dose required to induce severe disease. Here, we purified monkeypox virus with the goal of lowering the challenge dose by removing cellular and viral contaminants within the inoculum. We found that there are advantages to using unpurified material for intravenous exposures.

## 1. Introduction

As of the end of June 2022, there were close to over 4900 cases of human monkeypox spanning 50 countries that are non-endemic for the disease. Transmission has extended beyond cases linked to initial exposure in Africa to human-to-human spread in each country [1]. Because monkeypox virus does not transmit well and requires close contact, approved vaccines and therapies are available, and the circulating virus belongs to a less virulent monkeypox virus clade, known as the Western African clade, makes one surmise that the outbreak could be easily contained [1,2,3,4,5]. Recently approved smallpox and monkeypox therapeutics and vaccines are now getting real world application, and gaps in our countermeasure repertoire are being highlighted [6]. Therefore, it is appropriate to continue therapeutic and vaccine discovery and development of orthopoxvirus medical countermeasures to fill these gaps.

Because of the similarity of the diseases in humans and in the absence of an accessible etiological agent, MPXV is thought to be an appropriate surrogate for smallpox. Arguably, one of the premier models for severe human monkeypox and smallpox intervention is the intravenous monkeypox virus model in macaques using the Zaire 79 strain. To date, multiple vaccines and antivirals have been assessed in this model [7], including those licensed by the United States Food and Drug Administration (FDA) [8].

Delivery of monkeypox virus or variola virus is thought to mimic the secondary viremia in human monkeypox and/or smallpox disease. In macaques, similar to humans, fever is followed by the onset of a centrifugal rash. In classical disease, a maculopapular rash progresses to vesicles, pustules, and then umbilicates before scabbing. The main correlate of the intravenous model is death with secondary correlates being lesion counts and DNA viremia. Despite its popularity and faithfulness to lesional and fulminant orthopoxvirus disease, improvements such as delivery via a more natural route and/or decreasing the amount of virus required for severe disease are coveted. Currently, the only low dosed, nonhuman primate model using monkeypox virus is the common marmoset [9,10]. The model is thought to mimic the early stages of disease, incubation through to the onset of the rash phase, culminating in death.

Traditionally, monkeypox virus primarily used for challenge has been propagated and collected from cell culture, sonicated, and freeze/thawed to liberate cell-associated mature virus (MV) and consequently disrupt any extracellular virus particles present, with no additional purification steps. Zwartow, Westwood, and Appleyard showed with sucrose gradient purified vaccinia and rabbitpox virus stocks that impurity, such as viral hemagglutinin and cellular protein, could be dramatically reduced [11]. As they pertain to animal models, the impact of these impurities has never been directly evaluated. In theory, these extraneous impurities could serve to prime the immune system, thereby limiting infection. In fact, vaccination post smallpox exposure is thought to provide some benefit in limited studies [12,13]. Until recently, researchers had not tested alteration to the makeup of monkeypox viral inoculums in cynomolgus macaques (Mucker, in review at Viruses).

Here, we directly compare and characterize the disease induced by purified monkeypox virus, Zaire strain. We capture clinical manifestations and correlates of the model using two different doses of the monkeypox virus Zaire administered intravenously. We show there are differences in disease between the high doses of virus, especially in the consistency in clinical presentation.

## 2. Materials and Methods

### 2.1. Viruses, Cells, and Assays

#### 2.1.1. Virus and Propagation

Monkeypox virus strain Zaire was propagated from scab material on chorioallantoic membranes, followed by one passage in LLC-MK2 cells, two passages in BSC-40 cells, and two passages in Vero E6 cells. This material was received from the Center for Disease Control and Prevention and passed in MA104 cells. This virus will be referred to as the master stock and was provided by John Huggins (USAMRIID).

The master stock of the virus was thawed, sonicated, and vortexed four times. MA104 cells were inoculated at a multiplicity of infection (MOI) of 0.5 (5 plaque forming units for every 10 cells). The flasks were incubated in a 37 °C incubator and rocked every 15 min for 1 h. DMEM supplemented with 5% fetal bovine serum was added to each flask, after which the flasks were returned to the incubator. After 3 days, the cells were monitored for a cytopathic effect (CPE) daily. After a CPE of 4+ was noted, the supernatant was removed, cells were scraped and pooled, and three freeze-thaw cycles were performed. The material was then subjected to three sonication/vortex cycles and centrifuged to remove cells and larger cell debris. The supernatant was collected, and aliquots were made into pre-labeled tubes. This material will be known as the “crude” or “unpurified” working stock. A portion of this material was purified via a sucrose cushion, followed by a sucrose gradient [14], and was provided by Lisa Hensley (USAMRIID).

#### 2.1.2. Viruses Titrations

Monkeypox virus, to include the purified and crude inoculums, was titrated using a standard plaque assay [15]. One hundred microliters of sample was adsorbed onto at least two wells containing Vero-E6 cells (6-well dishes) for 1 h in a 37 °C incubator, with gentle rocking every 15 min. Two milliliters of EMEM containing 2% heat-inactivated FBS was added to each well and allowed to incubate for 4 days at 37 °C. The wells were then stained with a gentian violet solution (0.4% gentian violet) for at least 30 min, after which the stain was removed and the monolayers washed with distilled water. Plaques were subsequently enumerated.

### 2.2. Nonhuman Primates

#### 2.2.1. Conduct and Exposure

Adult, male Mauritius cynomolgus macaques (*Macaca fascicularis*) were screened for neutralizing antibodies to monkeypox virus prior to infection as described below. We opted to use cynomolgus macaques, since they tend to exhibit a more severe disease than do rhesus macaques and, as such, are more sensitive to the virus [14]. Physicals, lesion counts, and blood draws were performed prior to challenge, the day of challenge, and every two days post exposure until day 14. After which, day 17 and day 20 data (termination of the study) were collected. All exposures were performed using intravenous infusion via the saphenous vein. Maximum obtained lesion counts for each animal were grouped and statistically compared between respective groups using Student’s *t*-test comparisons (GraphPad Prism, San Diego, CA, USA). Necropsies were performed by USAMRIID’s Veterinary Pathology Division.

#### 2.2.2. NHP Neutralization Assays (Prescreening)

To confirm animals were not previously exposed to orthopoxviruses, neutralization assays were performed. Serum from animals was heat inactivated (56 °C for 30 min) and serially diluted in MEM alpha with 2% heat-inactivated FBS and HEPES. A target of 100 PFU/100 µL was added to each dilution and allowed to incubate at 4 °C overnight. Both positive (vaccinia immunoglobulin) and negative (media only) controls were concomitantly prepared. Titration of the samples was performed in duplicate wells using plaque assay. Results were reported as a percent reduction of the negative control.

#### 2.2.3. DNA Extraction and Quantitative PCR (QPCR)

Extractions and QPCR were performed as described previously [15]. Briefly, 100 µL of whole EDTA whole blood was extracted using a Qiagen DNA Blood Kit and eluted using 100 µL of buffer provided by the manufacturer. For QPCR, 5 µL of the eluted DNA was assayed in duplicate using the assay described in Kulesh et al. [16]. The averaged values were multiplied by 200 to yield genomes per milliliter. Maximum obtained values for each animal were statistically compared between groups using a Student’s *t*-test (GraphPad Prism).

#### 2.2.4. Chemistry and Hematology

Hematological data was generated on an ACT 10 Beckmann Coulter using whole EDTA blood. Abaxis Piccolos were used to evaluate clinical chemistries using Abaxis Chem12 or Chem13 reagent disks using serum samples.

#### 2.2.5. Necropsy, Histology, and Immunohistochemistry (IHC)

A necropsy was performed on all animals, either as soon as death occurred or after humane euthanasia of terminally ill or moribund animals. All tissues were immersion-fixed in 10% neutral buffered formalin for a minimum of 21 days, according to institute protocol.

Formalin-fixed tissues for histologic examination were trimmed, processed, and embedded in paraffin according to established protocols [17]. Histology sections were cut at 5 µm, mounted on glass slides, and stained with hematoxylin and eosin (H&E). Immunohistochemical staining was performed on replicate tissue sections using an Envision + kit (DAKO, Carpinteria, CA, USA). Normal tissue served as the negative control; the positive control was from a known monkeypox virus-infected nonhuman primate; and normal (uninfected) IgG was used as the negative control. Briefly, sections were deparaffinized in xyless, rehydrated in graded ethanol, and endogenous peroxidase activity was quenched in a 0.3% hydrogen peroxide/methanol solution for 30 min at room temperature. Slides were washed in PBS and then sections were incubated in the primary antibody, a non-commercial rabbit polyclonal antibody against vaccinia virus, diluted 1:3500 for 60 min at room temperature. Sections were washed in PBS and incubated for 30 min with Envision + rabbit secondary reagent (horseradish peroxidase-labeled polymer) at room temperature. Peroxidase activity was developed with 3,3′-diaminobenzidine (DAB), counterstained with hematoxylin, dehydrated, cleared with xyless, and then coverslips added.

## 3. Results

We hypothesized that by purifying MPXV, and thus reducing any confounding stimulation of the immune system by a contaminating material, we could effectively lower the lethal dose of the intravenous MPXV macaque model. To test this supposition, we intravenously exposed cynomolgus macaques (Mauritius) with two different target doses, 5 × 10^7^ PFU/mL (high dose) and 5 × 10^6^ PFU/mL (low dose) of sucrose gradient purified MPXV and our “crude” (unpurified or cell lysate) preparation. Historically, the high and low doses represent a near uniformly lethal and sublethal challenge, respectively [16,17,18,19].

### 3.1. Purified and Unpurified Innoculum Characterization

When the virus inoculums were back titrated (Table 1A), the purified groups were exposed to 3–5 times more virus than their respective crude counterparts (Table 1B). This was confirmed via QPCR of the inoculums (Table 1B). A direct comparison of genomes to PFU revealed a range of 6–27 (mean of 14.5) genomes/PFU, in line with previous findings [20]. However, purified monkeypox viral stocks used to prepare the inocula were plaque titrated multiple times to establish a titer (see Discussion), and animals receiving purified virus received more virus.

### 3.2. Survival

**Figure 1:**
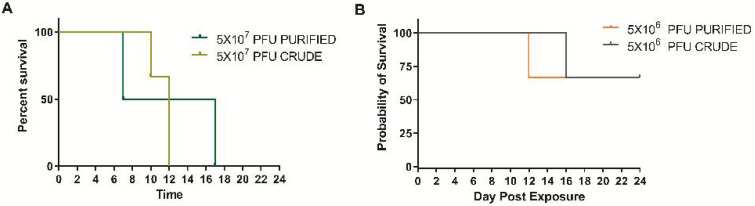
Percent survival in cynomolgus macaques intravenously exposed to either sucrose purified or traditional (crude) preparation at two concentrations of monkeypox virus. (**A**) The target dose for the high concentration was to mimic those used in viral therapeutic testing. (**B**) A 10-fold dilution of the high dose was utilized to determine any increased virulence (based on mortality) There were no significant differences (logrank test) in survival curves in either the high or low dose groups (*p* = 0.3213 and 0.8864, respectively).

The high dose of purified virus and crude virus produced a uniformly lethal disease. Animals in the purified group succumbed or were euthanized on days 7 and 17, whereas the crude virus group succumbed or were euthanized on days 10 and 12 (Figure 1A). Animal 2HP was euthanized for humane reasons on day 13 due to limb necrosis caused by extravascular leakage during exposure. The data for this animal will be discussed but has been removed from any statistical analysis. Both the purified and crude virus was 33% lethal, with animals being euthanized on days 12 and 16, respectfully (Figure 1B). There was no difference in survival curves using Graphpad’s logrank test within the high concentration or low concentration groups (*p* = 0.3213 and 0.8864, respectively).

### 3.3. Disease Course

Disease progression was similar in all groups with the exception of two animals from the purified high dose group (1HP and 2HP). Animal 1HP had an accelerated disease course, became confluent in a two-day time period, and subsequently died, whereas animal 2HP had a less severe disease state and had a 5 × 6 cm necrotic lesion at the site of exposure, indicating extravascular leakage during exposure. This animal was euthanized on day 13 for humane reasons. Lymphadenopathy was one of the earliest clinical signs of disease that developed between days 2 and 6 and peaked in size between day 6 and 14. During this peak time (days 6–14), a large portion of the animals became recumbent, dehydrated, and had a loss of appetite (Table 2). Less frequently, edema of the hands, feet, and head were noted. Nasal discharge was present in all unpurified high and one purified low dose animal. Lymphadenopathy was present in all animals. In survivors, the lymphadenopathy resolved late in the disease course.

**Figure 2:**
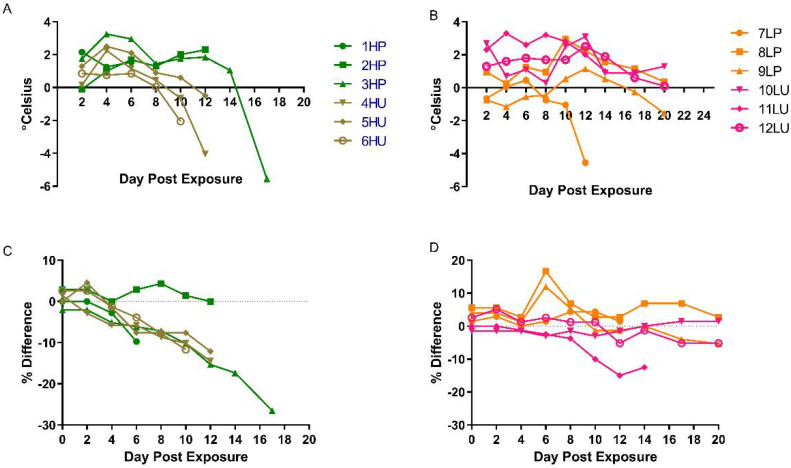
Temporal baseline-corrected temperatures and weights. Baseline-corrected temperatures (degree change from baseline) of cynomolgus macaques exposed to purified (high dose/purified = HP and low dose/purified = LP) or crude (high dose/unpurified = HU and low dose/unpurified = LU) monkeypox virus by individual animals; high dose (**A**) and low dose (**B**). Baseline-corrected (% difference) changes in weight of nonhuman primates intravenously exposed to monkeypox, using either purified or crude material at high (**C**) or low (**D**) concentrations of virus.

Animals in both high dose groups had elevated temperatures starting between days 2 and 4 (Figure 2A). The low dose groups were more variable, with some temperature increases occurring early (days 2–4), late (days 10–12), or not at all (Figure 2B). Temperatures tended to drop towards terminal points in the disease. Weight also decreased as much as 26%, 15%, 7%, and 15% in the high purified, high crude, low purified, and low crude dose groups, respectively (Figure 2C,D). Not all animals that were terminal experienced a decrease in weight, as animal 7LP exhibited little change throughout the disease course (Figure 2). Based on the parameters discussed, there were no obvious differences in disease between purified and crude preparations.

### 3.4. Lesion Burden and Progression

In general, lesions developed in all animals between days 4 and 6 with the entire crude high dose cohort developing lesions on day 4 and the remainder on day 6 (Table 3). With the exception of three animals, rash progressed in a typical fashion from macular/papular rash to vesicular/pustular/umbilicated lesions, and eventually scabs (Table 3). Vesicles were the last stage of lesion development for animal 1HP, as it died on day 7 before the disease could progress. Two other primates, 6HU and 7LP, never developed scabs (Table 3). Of the 12 nonhuman primates, 6 had confluent lesions (Table 3) that were primarily located on the hands, feet, and head. Five of these animals were in the highest dose groups (Table 3).

**Figure 3:**
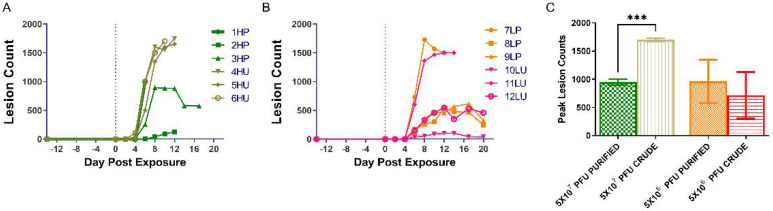
Temporal and total lesion counts for nonhuman primates exposed to either purified or unpurified preparations of monkeypox virus. Lesions were quantified and categorized every 2nd day post exposure in one of four groups: Macular/papular/vesicular, pustular/umbilicated, scabbing, and desquamating. The individual lesion counts per day for the high dose groups and the low dose groups and presented in (**A**,**B**), respectfully. The maximum number of lesions for each animal were combined per the individual’s respective group (**C**). A two-tailed *t*-test was performed between the two high dose groups and between the two lower dosed groups. A statistical difference was noted when comparing the maximum lesion counts in the high dose groups (*p* = 0.0008) and is represented by the asterisk. HP, high dose purified; LP, low dose purified; HU, high dose crude; LU, low dose crude.

The numbers of lesions (peak lesions) were quite different between the high dose purified and unpurified groups and quite variable between animals in the low dose groups (Figure 3A,B). The ranges for the peak lesion counts were as follows: 538–1729 and 104–1500, low dose purified and unpurified; 895–1000 (animal 2HP was not included and had a peak lesion count of 126 lesions) and 1650–1750 high dose purified and unpurified (Figure 3C). Statistically, there was a significant difference in the peak number of lesions between the high dose groups (*p* = 0.0008). There was no difference between the low dose groups (*p* = 0.7542). The difference and variability of lesions in the high dose groups suggest an advantage for utilizing crude preparations of virus for intravenous nonhuman primate exposures.

### 3.5. Viral Genomic Burden

After exposure, blood was immediately (within 2 min) sampled to confirm exposure and consistency among animals within a group. Animals in three of the four groups were consistently challenged, with ranges of 4.5 × 10^4^ to 7.2 × 10^4^ genomes/mL and 5.6 × 10^5^ to 7.5 × 10^5^ genomes/mL for the crude low and high groups and 1.0 × 10^5^ to 1.5 × 10^5^ genomes/mL for the low dose purified group. The high dose purified group had a little more variability in one animal (2HP) with values of 2.1 × 10^6^, 2.3 × 10^6^, and 1.9 × 10^4^ genomes/mL. This animal had evidence of extravascularization of the virus (see disease course and pathology sections), and this would explain the difference in circulating genomes. The crude and purified high dose groups were approximately 10-fold higher than the respective low dose purified and unpurified groups. Similar to the inoculums, the purified groups were higher than the crude counterparts.

**Figure 4:**
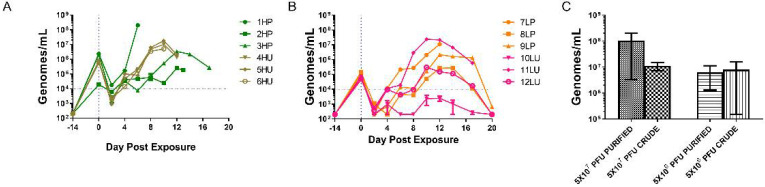
Viremia as assessed via quantitative polymerase chain reaction of EDTA whole blood. Evaluation of the genomic viral loads were conducted every two days (and at euthanasia, when possible) for the high dosed (**A**) and low dosed (**B**) monkeypox virus exposed groups. The peak values obtained for individual animals were combined per group (**C**), and the log_10_ transformed values were statistically evaluated using a two-tailed *t*-test between the two high dose groups and between the two lower dose groups. There was no statistical difference between either the high (*p* = 0.3348) and low (*p* = 0.5001). HP, high dose purified; LP, low dose purified; HU, high dose crude; LU, low dose crude.

Viral genomes were detected as early as day 2 in all groups, with only one of the samples (animal 1HP) being quantifiable (above 50 gen/reaction or 10,000 gen/mL) (Figure 4). All high dose-exposed nonhuman primates had quantifiable samples by day 4. The days ranged between 4 and 10 when values were quantifiable in the low dose group, and in one case, 10LU; quantifiable genomes were never achieved. In all cases, values increased and peaked between days 8 and 12, and survivors gradually decreased to nominal values by day 20.

As with the lesion counts, blood Q-PCR values varied amongst the purified high dose and both low dose groups (Figure 4A,B). The highest level, 2 × 10^8^ genomes/mL, was from the high dose purified group, animal 1HP, reflecting its accelerated disease course (see disease development and lesion burden). The other two members of this cohort had a peak viral DNA load 2–3 logs lower. The crude, high dose group was much more consistent and ranged from 5 × 10^6^ to 2 × 10^7^ genomes/mL. These quantities were obtained between days 6 and 12 with all crude maximums falling on day 10 (Figure 4B). It is important to note that the animal with the lowest peak genome value (animal 2HP) also had the lowest value immediately after infection.

The peak genome levels for low dose groups ranged from 3 × 10^5^ to 1 × 10^7^ genomes/mL and 2 × 10^3^ to 2 × 10^7^ genomes/mL for purified and crude, respectively, and occurred at days 10 or 12 (Figure 4C). All crude low dose peak QPCR values were captured on day 10, whereas all purified low dose values peaked on day 12 (Figure 4A,B). There was no significant difference when the maximum values between similarly dosed groups were compared (high dose, *p* = 0.3348 and low dose, *p* = 0.5001). In summary, comparison of QPCR values in animals receiving the high dose of monkeypox virus suggests that consistency might be an advantage for using crude preparations.

### 3.6. Hematology and Chemistry

**Figure 5:**
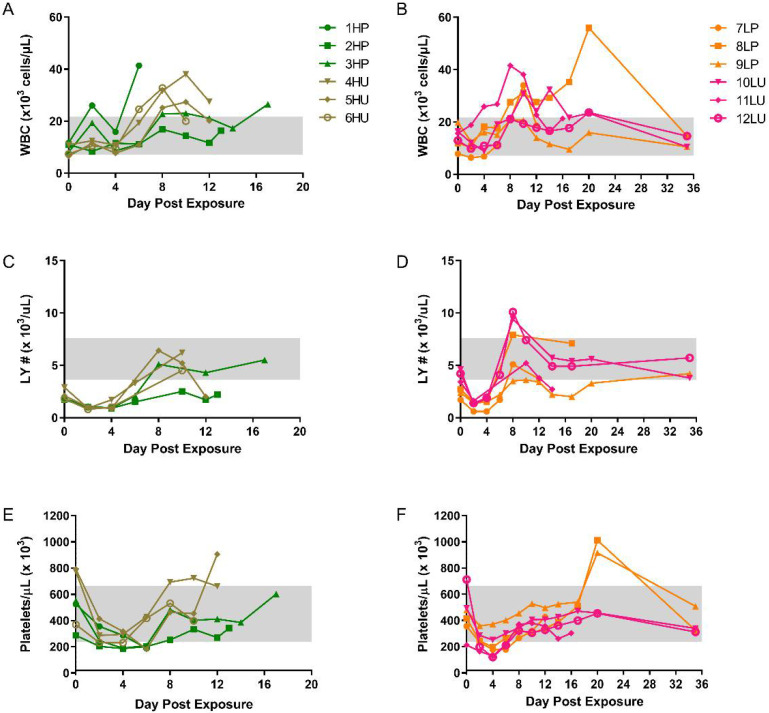
Temporal hematological changes in cynomolgus macaques intravenously exposed to either purified or unpurified monkeypox virus at two different doses. Whole EDTA blood was run on the Beckman Coulter AcT 10. Absolute white blood cell counts (WBC) (**A**,**B**), lymphocyte number (LY#) (**C**,**D**), and platelets (**E**,**F**) are shown. HP, high dose purified; low dose purified, LP; high dose crude, HU; low dose crude, LU. Normal ranges (grey boxes) were previously reported [21], except figures (**C**,**D**).

Increases in circulating white blood cells (WBC) were noted for all groups, with most animals peaking between days 8 and 12 (Figure 5A,B). Animal 1HP was one of the few exceptions, with a peak value occurring a day before succumbing to disease (day 6). Two others succumbed on day 18 and 20 from animals in the high (3HP) and low (8LP) dose purified group, respectively. After peaking, survivors slowly returned to near baseline levels. Similar onset and trends were noted for lymphocytes in all groups, with all but one peaking between days 8 and 10 post exposure (Figure 5C,D). Platelet counts slightly decreased in all groups between days 2 and 4 post exposure (Figure 5E,F). This was followed by an increase at or above baseline levels. No other remarkable hematological features (RBC, HCT, MCV, MCH, MCHC) were noted. These general trends are consistent with historical data from this model [13].

**Figure 6:**
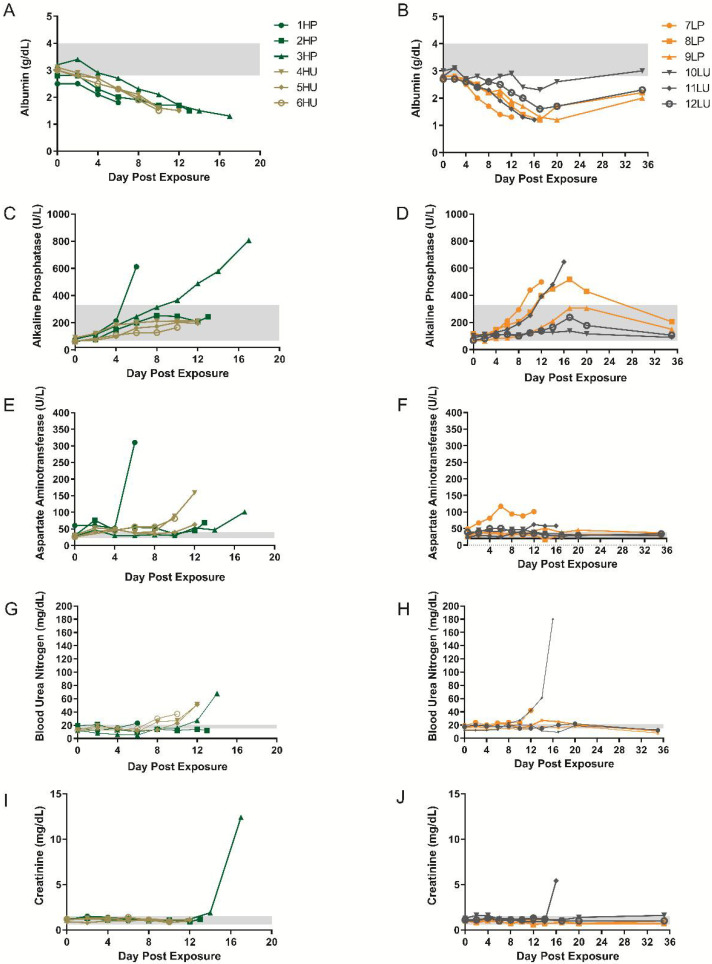
Blood chemistry analysis from cynomolgus macaques exposed to purified or unpurified preparations of monkeypox virus. Blood was collected into serum separating tubes (SST) and the resulting sera were analyzed using General Chemistry 12 discs using an Abaxis Piccolo. Analytes include albumin (**A**,**B**), alkaline phosphatase (**C**,**D**), aspartate aminotransferase (**E**,**F**), blood urea nitrogen (**G**,**H**), and creatinine, (**I**,**J**). HP, high dose purified; low dose purified, LP; high dose crude, HU; low dose crude, LU. Normal ranges (grey boxes) were previously reported [21], with the exception of (**E**,**F**).

Clinical chemistries were also performed. Albumin levels decreased starting between days 2 and 4 in the high dose groups and never recovered (Figure 6A). In comparison, animals in the low dose groups decreased post day 4, and, with the exception of 10LU, with a similar magnitude (Figure 6B). Survivors (low dose group) rebounded to near basal levels on the final collection day. Both high dose and low dose groups had an increase in alkaline phosphatase levels starting 2–4 and 4–12, respectively (Figure 6C,D). The highest values were obtained in the high dose purified group (1HP and 3HP) followed by three animals from the low dose groups, one of which survived, and as with the other survivors, returned to near basal values. AST and BUN values varied in magnitude but were at least slightly elevated at the time animals succumbed or were euthanized (Figure 6E–H). Elevations in creatinine were observed in two animals, 3HP and 11LU, late in the disease (Figure 6I,J). Chemistry and hematological data suggest no advantage of using one inoculum over the other.

### 3.7. Pathology

Gross necropsy findings included maculopapular rash and necrotizing and proliferative lesions of oral mucosa, lips, nares, tongue, trachea, esophagus, and lung in all groups. Additionally, lymphadenopathy of the axillary, inguinal, and tracheobronchial lymph nodes in both dose groups was present.

Histologically, the skin was the most consistently affected tissue among both groups. Epithelium of multiple tissues (e.g., oral mucosa, esophagus, trachea, and lung) exhibited multifocal proliferative and necrotic lesions. These lesions contained epithelial degeneration and necrosis with intracytoplasmic and intercytoplasmic edema and neutrophilic exocytosis as well as proliferation of epithelium at the periphery of the lesions. Syncytial cells and intracytoplasmic inclusion bodies (ICIB) were also present. Perivascular dermatitis of the lips and nares was noted in all animals except 4HU. Hemorrhage, mainly associated with the oral mucosa, was noted in 4 of 8 animals.

There was fibrinonecrotic pleuropneumonia in all animals excluding 1HP and 2HP. In affected animals, the upper and lower respiratory tract and pleura (trachea, bronchi, bronchioles, alveoli, and pleura) had multifocal necrosis of epithelium, alveolar septae, and pleural mesothelium with multifocal loss of architecture. Lesions contained mixed inflammatory cells, necrotic and cellular debris, fibrin, and hemorrhage. The pulmonary pleura was expanded by mixed inflammatory cells and abundant fibrin and occasional hemorrhage. Hepatocellular lesions were mild, with necrosis and degeneration in 4 of 8 animals. Three animals lacked hepatocellular damage (6HU, 2HP, and 7LU).

Focal proliferative and necrotic lesions with occasional hemorrhage were observed in the gastrointestinal tract (stomach, duodenum, and colon) of four animals. Animal 1HP also had multifocal epicardial petechial hemorrhage of the right and left ventricle. These lesions are uncommon in cynomolgus macaques intravenously exposed with monkeypox virus.

Additional histologic findings included plasmacytosis in the spleen (7/8), lymphoid depletion of multiple lymphoid tissues such as spleen and various lymph nodes (3 crude high dose, 1 purified high dose), hemorrhage (2/8), and congestion (3/8).

Bacteria were thought to play a role in some of the lesions seen. At the extreme, animal 1HP was bacteremic with colonies found in the bone marrow, liver, spleen, lung, and lymph nodes. Other examples of these lesions were associated with the urinary bladder of 11LU, colon of 3HP, and the axillary and inguinal LN of 4HU.

## 4. Discussion

The intravenous monkeypox model is the most utilized nonhuman primate model for the evaluation of vaccines and antiviral drugs developed to mitigate the potential reintroduction of variola virus into an immunologically naïve population [17,18,19,22,23,24,25,26,27,28,29,30,31,32,33,34,35,36,37,38]. The major criticisms regarding this model include the high titer required to elicit severe disease and the unnatural route of exposure, which bypasses early events (incubation) in human smallpox and monkeypox disease. These criticisms have prompted the optimization of the model.

In these studies, we sought to lower the lethal dose of monkeypox virus intravenously delivered to cynomolgus macaques by manipulating the starting material (inoculums). In our first series of experiments, animals were exposed to sucrose gradient purified monkeypox virus at two different doses and compared to virus preparation that had been historically utilized in this test system [3]. We hypothesized that by reducing extraneous viral and cellular contaminants, a near 100% lethality could be achieved using a reduced input of virus. We can reject this notion, as the high and low dose purified material produced similar lethality. With that being said, it is important to note that the purified groups received 3–5 fold more virus than the crude dose animals, and based on this, we would have expected higher mortality and more severe disease in the purified low dose group. For instance, 2 × 10^7^ PFU of intravenously administered unpurified monkeypox virus was 83% lethal in mock vaccinated cynomolgus macaques [26] and similar results were found in the slightly less susceptible rhesus macaque of 100% [37] and 80% [36].

The disease in all groups progressed in a fashion consistent with those previously reported and with features consistent with monkeypox or smallpox in humans [39,40]. A cutaneous rash progressed and was centrifugally distributed amongst all exposed animals. Although the onset of these lesions was noted earlier (day 4 vs. day 6) for the high dose crude group, this study was not designed to determine the statistical relevance. Furthermore, these are still congruent with onset observed by others [7]. The number of lesions was greater in animals that were euthanized or succumbed to disease than those that survived exposure and, with the exception of a single animal in the low dose purified group, tended to have confluent lesions. These attributes are normally associated with severe smallpox disease, clinically defined as ordinary type-confluent, and have a mortality rate of approximately 50–75%. Viremia, as determined via PCR, to some degree also reflected the outcome of disease, as animals with the highest viral load succumbed to disease.

In terms of disease treatment, rash or lesion onset is typically considered the first indicator of poxvirus disease and the trigger to start treatment. In our study, lymphadenopathy was consistently observed in all animals regardless of outcome, was one of the earliest clinical manifestations, and preceded rash in some cases. Peripheral lymphadenopathy is also a feature of the hemorrhagic and lesional variola model in cynomolgus macaque [41]. When testing antivirals for the treatment of monkeypox disease, using the monkeypox virus intravenous model, lymphadenopathy, in conjunction with qPCR, could be considered as a trigger to treat, with some caution. Swollen peripheral lymph nodes are a distinguishing feature of human monkeypox cases but are not reported for smallpox, and therefore, this treatment strategy may not fit a real-world scenario for smallpox disease.

In this report, chemistry and hematology reflected those reported for human monkeypox and/or smallpox disease [39]. Increases in leukocytes and thrombocytopenia have been reported for human cases of both monkeypox and smallpox disease. Chemistries generally did not directly predict the underlying pathological condition. As an example, a decrease in albumin and increases in alkaline phosphatase and aspartate aminotransferase suggest hepatic impairment, but only 4 of the 8 animals had significant hepatic findings, which were mild. As such, changes in these analytes are most likely due to nonspecific changes. For instance, a decrease in albumin is likely the effect of epithelial breakdown and lack of fluid homeostasis.

Two animals in the high purified dose group exhibited disparate disease manifestations, in the context of the dose administered. Animal 2HP presented a very mild disease course with reflective pathology. As previously stated, this animal most likely did not receive the full inoculum, as evidenced by the necrotic dermal lesion at the injection site and the relatively low circulating genomic load determined immediately after exposure. This animal was humanely euthanized as this lesion would have progressed and caused complications such as a severe secondary bacterial infection. The other animal, 1HP, progressed very rapidly, and succumbed to infection on day 7. Lesions never progressed beyond the vesicular phase. Again, this is also reflective of the pathological findings, where lymph nodes showed no evidence of an immunological response such as hyperplasia and plasmacytosis. This was most likely a consequence of its abbreviated disease course. Lesions not typically seen in the intravenous cynomolgus model were also present, such as those related to the heart. In fact, virally induced cardiac conditions have rarely been reported for monkeypox or smallpox afflicted individuals, but myocarditis, pericarditis, and ischemic cardiac events have been observed with live vaccines that are administered for protection against these diseases [42,43,44,45,46].

Consistency in an animal model system is important when evaluating therapeutic interventions. This is amplified when evaluating correlates of disease that are not absolute, as interventions may protect from the lethal aspect of the disease but may less effectively reduce other manifestations such as the onset or magnitude of the rash and viremia. In our study, there were no differences in mortality, although a few parameters had either significant changes or were more variable in nature. For instance, the unpurified high dose group exhibited a statistically higher mean peak lesional burden than the purified group. Because one of the animals (1HP) died early in the disease course, we cannot be certain it reached its “lesional potential”. Statistical analysis of lesion counts when analyzing only the lesion counts from day 6 did not produce any significance, but day 8, where the last lesion count (maximum value) for 1HP was carried forward, were significant (data not shown). There was also greater variability in time-to-endpoint when comparing the purified high dose group to the unpurified group (7 and 17 vs. 10 and 12, respectively). Reports in cynomolgus macaques have ranged from as early as 5 days [38] to as late as 18 days [17] with a majority of animals succumbing or euthanized around day 12 [16,18,19,27,28]. The variation between these reports could be attributed to differences in euthanasia criteria or the source or subspecies of *Macaca fascicularis*. Although we highlighted some potential differences, more animals would be required to reliably state that these are true differences. If real, these differences could be related to the relative variability between individual doses administered to the NHPs. It is likely that purification of the virus increases aggregation of virions and prevents equal distribution of the virus within the inoculum, and this leads to animals receiving different effective (and infective) doses. Given the sharp changes in disease manifestation based on dose [47] and reviewed in [7]), we will further elaborate on this point later in the discussion.

Our data suggest that purified monkeypox virus does not increase virulence as measured by mortality in the intravenous macaque model. Whether purification would alter other nonhuman primate models, such as those using a respiratory route of challenge, is still up for debate. Estep et al. exposed rhesus macaques to purified monkeypox virus via the intrabronchial route and compared the immunological response to a recombinant virus lacking the monkeypox inhibitor of the complement enzyme (MOPICE) gene (D14L) [48]. Of the wild-type exposed animals, 0 of 4 animals succumbed to infection when dosed with 2 × 10^5^ PFU/mL and 100% when dosed with approximately one and two more logs of virus. Animals succumbed to disease around day 10. The closest crude virus comparison is with the Johnson et al. study where cynomolgus macaques were exposed via a similar route and doses of 5 × 10^5^ and 5 × 10^6^ PFU/mL [47]. At these doses, 1 of 6 animals and 2 of 3 animals succumbed to disease at day 9 and days 19–21, respectively. It is difficult to compare the studies due to differences in host species and potential differences in euthanasia criteria, but, in general, the purified material in the rhesus macaque seemingly had a more rapid disease course and was more lethal. It has been suggested through retrospective analysis that rhesus macaques are less sensitive to monkeypox virus [7]. If true, it would strengthen the case for a difference between purified and crude virus. But again, a head-to-head comparison would still need to be performed.

There are advantages to using a crude virus, such as a potential decrease in virion aggregation. Continued use of purified stocks will entail characterization that was already conducted for unpurified stocks. In fact, titer calculation for our purified stock tended to change with increasing freeze-thaws and sonication. The final stock values were calculated after consistent values were established independently by two individuals and were later still unreliable as the animals received more virus than originally calculated via plaque assay (Table 1). An underestimate of the stock titer was also confirmed by our more reliable qPCR assay [20]. Although crude stocks still require sonication before use to break up aggregating virions, the results are much more accurate (Table 1). As stated earlier, aggregation would also explain the disease variability seen in animals receiving a high dose of purified material. That is, even though all animals in this group received virus from a single preparation, it is possible that animals may have received a less (or more) “effective dose” depending on the amount of aggregation in the specific syringe. With that in mind, purified stocks may be more applicable when specific immunological responses and/or response mechanisms are being evaluated. Using purified stocks, Rubins et al. showed that monkeypox virus elicited little innate response in vitro, unlike inactivated monkeypox virus [49]. The incorporation of extraneous antigen, like that found in crude virus, could alter this proposed stealth strategy proposed for monkeypox virus and produce different results. For purposes of dissemination via an intracellular mechanism, attracting certain trafficking cell types might be advantageous for monkeypox virus. Which set of conditions is most applicable to human infection with variola virus or monkeypox virus is anyone’s guess, but further examination, similar to ours, should be considered.

## Figures and Tables

**Table 1:** Comparison of purified and unpurified monkeypox virus inoculums.

(**A**)			
**Empirical Dose:**			
**Target Dose**	(PFU/mL) *	**PCR (gen/mL)**	
5 × 10^7^ pure	1.8 × 10^8^	2.35 × 10^9^	
5 × 10^6^ pure	1.4 × 10^7^	3.75 × 10^8^	
5 × 10^7^ crude	6.6 × 10^7^	7.90 ×10^8^	
5 × 10^6^ crude	3.1 × 10^6^	1.85 × 10^7^	
**(B)**			
**Target Dose**	**GEN to PFU Ratio**	**Fold Difference** **between Respective Dosing Group (by Plaque Assay) ^1^**	**Fold Difference between Respective Dosing Group (by QPCR) ^2^**
5 × 10^7^ pure	13	2.7	3.0
5 × 10^7^ crude	12	-	-
5 × 10^6^ pure	27	4.6	20
5 × 10^6^ crude	6	-	-

* Plaque assay data from two users titrating independently were used to generate values. ^1^ Value obtained by dividing back titrated inoculums by comparable dosing group. ^2^ Values obtained by dividing qPCR values by comparable dosing group.

**Table 2:** Clinical signs and onset in cynomolgus macaques intravenously exposed to monkeypox virus by day of onset *.

	Lymphadenopathy	Recumbency	Decrease in Appetite	Dehydration	Edema	Nasal Discharge
High Dose Purified	2, 2, 4	4, 4, N/A	6, 6, 8	10, 6, 4	17, N/A, 8	N/A, N/A, N/A
High Dose Crude	4, 4, 4	8, 6, 4	6, 6, 6	6, 6, 8	12, 8, N/A	8, 8, 8
Low Dose Purified	4, 4, 2	6, 8, 14	N/A, N/A, N/A	4, 4, 8	12, N/A, N/A	N/A, N/A, N/A
Low Dose Crude	6, 4, 4	N/A, 12, 14	N/A, 6, 6	N/A, 10, 10	N/A, 12, 12	N/A, 8, N/A

* By individual nonhuman primate. N/A = condition not observed.

**Table 3:** Key features of the onset and progression of rash, by animal and day of onset.

Group	Lesion/Rash	Pustule/Umbilicated	Scab	Confluent Lesions (# of NHPs)
High Dose Purified	6, 6, 6	8, 8, N/A	10, 8, N/A	2/3
High Dose Crude	4, 4, 4	8, 8, 8	12, 12, N/A	3/3
Low Dose Purified	6, 6, 6	8, 8, 10	8, 8, 12	0/3
Low Dose Crude	6, 6, 6	N/A, 8, 8	8, 8, 8	1/3

N/A = condition not observed.

## Data Availability

Data is contained within the article or Mucker et al. (Viruses, in review) [50].
